# Lessons Learned from Experimental Human Model of Zinc Deficiency

**DOI:** 10.1155/2020/9207279

**Published:** 2020-01-09

**Authors:** Ananda S. Prasad

**Affiliations:** Department of Oncology, Wayne State University School of Medicine and Barbara Ann Karmanos Cancer Center, Detroit, Michigan 48201, USA

## Abstract

Zinc is an essential element for humans, and its deficiency was documented in 1963. Nutritional zinc deficiency is now known to affect over two billion subjects in the developing world. Conditioned deficiency of zinc in many diseases has also been observed. In zinc-deficient dwarfs from the Middle East, we reported growth retardation, delayed sexual development, susceptibility to infections, poor appetite, and mental lethargy. We never found a zinc-deficient dwarf who survived beyond the age of 25 y. In an experimental model of human mild zinc deficiency, we reported decreased thymulin (a thymopoietic hormone) activity in Th1 cells, decreased mRNAs of IL-2 and IFN-gamma genes, and decreased activity of natural killer cells (NK) and T cytotoxic T cells. The effect of zinc deficiency on thymulin activity and IL-2 mRNA was seen within eight to twelve weeks of the institution of zinc-deficient diet in human volunteers, whereas lymphocyte zinc decreased in 20 weeks and plasma zinc decreased in 24 weeks after instituting zinc-deficient diet. We hypothesized that decreased thymulin activity, which is known to proliferate Th1 cells, decreased the proliferation differentiation of Th1 cells. This resulted in decreased generation of IL-2 and IFN-gamma. We observed no effect in Th2 cell function; thus, zinc deficiency resulted in an imbalance of Th1 to Th2 function resulting in decreased cell-mediated immunity. Zinc therapy may be very useful in many chronic diseases. Zinc supplementation improves cell-mediated immunity, decreases oxidative stress, and decreases generation of chronic inflammatory cytokines in humans. Development of sensitive immunological biomarkers may be more sensitive than an assay of zinc in plasma and peripheral blood cells for diagnosis of marginal zinc deficiency in human.

## 1. Introduction

Raulin in 1869 [1] showed for the first time that zinc was required for the growth of Aspergillus niger. Essentiality of zinc for growth of plants in 1926 and the growth of rats in 1934 was reported [2, 3]. In 1955, a disease called parakeratosis in swine due to zinc deficiency was observed [4]. Zinc was shown to be essential for chickens in 1958 [5]. In animals, the manifestations of zinc deficiency included growth failure, loss of hair, thickening and hyperkeratinization of the epidermis, and testicular atrophy. Deficiency of zinc in breeding hens resulting in decreased hatchability, gross embryonic anomalies characterized by abnormal skeletal development, and weakness in chicks that hatched were observed [6]. Although the essentiality of zinc for animals was established, its ubiquity made it seem improbable that zinc deficiency in humans could lead to significant problems in humans. During the past 50 years, however, it has become apparent that deficiency of zinc in humans is quite prevalent.

I went to Shiraz, Iran, in 1958. I had finished my training as a Medical Scientist under the guidance of Prof. C.J. Watson. I accepted an invitation from Prof. H.A. Reimann to help him set up a medical curriculum and training program at the Shiraz Medical School, Shiraz, Iran.

Within three weeks of my arrival in Shiraz, my Chief Resident showed me a 21 yr old male who was extremely growth retarded, had no secondary sexual characteristics, and had hypogonadism, hepatosplenomegaly, rough skin, and mental lethargy [7]. He was severely anemic, and the anemia was due to iron deficiency. There was no blood loss. Severe iron deficiency in males does not occur without loss of blood. His diet consisted of only bread made of unleavened flour, and animal protein intake was negligible.

Iron deficiency does not cause growth retardation and hypogonadism. An examination of the periodic table suggested to me that perhaps this syndrome was due to both iron and zinc deficiency [7]. We suggested that perhaps high content of phosphate in the diet and geophagia, which was very common in villagers of Iran, may have impaired absorption of both iron and zinc. We studied 10 additional cases in Nemazee Hospital, Shiraz, Iran, within a short period of time, suggesting that perhaps zinc deficiency in the Middle East may be common.

I subsequently went to US Naval Medical Research Unit No. 3 in Cairo, Egypt, and studied zinc metabolism extensively in Egyptian dwarfs.

We were able to document that growth retardation and hypogonadism in these subjects were due to zinc deficiency. Zinc concentration in plasma, red cells, urine, and hair was decreased; the Zn 65 plasma turnover rate was increased, and the 24 h exchangeable pool of Zn 65 was decreased in all the dwarfs [8, 9].

In Egypt, the rate of growth was greater in patients who received supplemental zinc as compared with those receiving iron instead or those receiving only an adequate animal protein diet. Pubic hair appeared in all subjects within 7-12 wk after zinc supplementation. Genitalia size increased to normal size, and secondary sexual characteristics developed within 12-24 wk in all subjects following zinc supplementation. In contrast, no such changes were observed in a comparable length of time in the iron-supplemented group or in the group on an animal protein diet alone [10]. The growth retardation and gonadal hypofunction in these subjects were related to zinc deficiency. The anemia was due to iron deficiency and responded to oral iron treatment. These studies clearly showed that severe anemia and iron deficiency were not causative factors for growth retardation and hypogonadism in these subjects.

The diet of the Middle Eastern subjects (both Iran and Egypt) consisted of mainly cereal protein high in phytate (an organic phosphate compound) which complexes both zinc and iron resulting in decreased availability of both zinc and iron.

We concluded that excess content of dietary phytate was responsible for deficiencies of both iron and zinc in Middle Eastern dwarfs [7, 8, 10].

The recommended dietary allowance for zinc was established in 1974 by the US National Academy of Science, National Research Council [11]. The current estimate of WHO is that nearly two billion subjects in the developing world may have nutritional deficiency of zinc.

Childhood malnutrition is a common problem in the developing world. A 1993 report based on WHO's global data of children's growth showed that approximately 42% of children less than 5 y of age have low height compared to the international reference standard [12, 13].

The causes of growth failure are not fully understood; inadequate food nutrition and poor nutritional quality of many traditional foods may be important factors.

The high intake of cereal protein rich in phytate prevents absorption of zinc and iron. Zinc is extremely important for growth, and our studies in the Middle East in the sixties showed that the dwarfs had adequate intake of caloric and vitamins but were zinc deficient [7–10].

There have been several studies, which document the effect of zinc supplementation on increase in height and gain in weight in children who were supplemented with zinc.

Brown et al. [13] published a meta-analysis of 33 studies published on the effect of zinc supplementation in children.

There was a highly significant effect of zinc supplementation on increase in height [13]. These studies indicate that zinc deficiency is a global health problem, which affects growth and development in children.

It is now evident that nutritional as well as conditioned deficiency of zinc may complicate many diseases in human subjects.

## 2. Zinc Deficiency in Human

### 2.1. Severe Zinc Deficiency

A severe deficiency of zinc may be life-threatening as has been reported in patients with acrodermatitis enteropathica (AE) [14] (a genetic disorder), after use of total parenteral nutrition without zinc, after penicillamine therapy, and with acute alcoholism. The clinical manifestations of severely zinc-deficient subjects include bullous-pustular dermatitis, diarrhea, alopecia, mental disturbances, and intercurrent infections due to cell-mediated immune deficiency; if untreated, the zinc deficiency becomes fatal. AE is caused by a mutation in a zinc transporter, ZIP-4, which results in malabsorption of zinc [15].

### 2.2. Moderate Level of Zinc Deficiency

Growth retardation, hypogonadism, skin changes, poor appetite, mental lethargy, abnormal dark adaptation, and delayed wound healing are some of the manifestations of moderate zinc deficiency in human subjects. Moderate deficiency of zinc due to nutritional factors, malabsorption, sickle cell disease, chronic renal disease, and other debilitating conditions has now been well documented [7–10].

During my stay in the Middle East, I never saw a dwarf older than 25 years of age. Local physicians told us that the dwarfs died because of infections, suggesting that zinc deficiency may have affected their immunity adversely.

Females are also affected by zinc deficiency. Although this was not appreciated in earlier studies, later studies documented zinc deficiency in females. Growth retardation and ovarian dysfunction due to zinc deficiency were observed in zinc-deficient females [16].

### 2.3. Marginal Deficiency of Zinc

A mild zinc deficiency was induced by an experimental soybean-based protein diet in normal human volunteers. The diet met all recommended dietary allowances for protein and essential macro- and micronutrient except for zinc. The daily intake of zinc was restricted to 3 to 5 mg daily. The RDA for zinc in adults is 12-15 mg/d. [16, 17].

The volunteers were admitted to a Clinical Research Center metabolic ward at the University of Michigan Hospital. They were ambulatory and encouraged to do daily exercises. They were studied for a total of 24 weeks.

The details of our protocol have been published [17].

We observed neurosensory changes, oligospermia in males, decreased serum testosterone concentration, hyperammonemia, decreased lean body mass, decreased serum thymulin activity, decreased IL-2 activity, decreased NK cell activity, and alterations in T cell subpopulations in our marginally zinc-deficient subjects. All the above manifestations were correctable by supplementation with zinc [17–19].

## 3. Zinc and Immunity

Bach et al. [20] described the biochemical characteristics of a thymic hormone protein called facteur thymique serique (FTS). This was later called thymulin. In 1983, it was reported that zinc was essential for its activity [21].

At that time in Detroit, we were working with a human experimental mild zinc deficiency model. The reports from France were very exciting. Our studies in the Middle East showed that the dwarfs were susceptible to infections, and they died before they reached 25 years of age. The thymulin zinc study suggested to me that zinc was involved in immunity. The report that a thymic hormone required zinc for its activity stimulated me to collaborate with Prof. Bach and Dardene.

I met them in Paris, and they were equally excited to study thymulin in a human zinc deficiency model. Together, we published several papers on these subjects. We observed that thymulin was required for T helper cell differentiation and proliferation. We reported that zinc was required for gene expression of IL-2 and IFN-gamma [18]; IL-2 generation upregulated natural killer cell activity and T cytotoxic cell activity [18, 19, 22]. IFN-gamma along with IL-12 generated by monocytes-macrophages was important for the activity of monocytes-macrophages for killing viruses, bacteria, and parasites.

In mild deficiency of zinc in the human model, we observed decreased serum thymulin activity, which was corrected by in vivo and in vitro zinc supplementation, suggesting that this index was a sensitive indicator of mild zinc deficiency in humans [18]. It is probable that because of zinc deficiency, host-defense mechanisms were compromised in a large segment of population in the developing world.

The effect of zinc on lymphocytes also appears to be that of a mitogen, and the kinetics of these influences most closely approximate the effects of antigen stimulation on lymphocyte in culture [23]. It is suggested that zinc directly stimulates DNA synthesis, either by activating enzymes or by altering the binding of F1 and F3 histones to DNA, which affects RNA synthesis. Direct cell-surface effects of zinc cannot be ruled out. It is conceivable that zinc could be operating at several different levels in influencing lymphocyte monoclonal proliferation [23].

Iwata et al. in 1979 [24] showed that one obvious effect of zinc deficiency was decrease in thymocytes in mice and humans which resulted in reduction of thymic hormone activity.

DNA polymerase, RNA polymerase, reverse transcription, and deoxythymidine kinase are zinc-dependent enzymes and are involved in DNA synthesis [25].

We reported that zinc is required for the gene expression of deoxythymidine kinase, an enzyme essential for DNA synthesis and cell division [26–29].

## 4. Nucleoside Phosphorylase (NPase) and Zinc

Giblett et al. in 1975 [30] reported a case of a 5 y old girl with severely defective T cell immunity, normal B cell, and nucleoside phosphorylase deficiency.

Nucleoside phosphorylase catalyzes the conversion of inosine and deoxyinosine to hypoxanthine and guanosine and deoxyguanosine to xanthine. Accumulation of deoxyguanosine is believed to be toxic to T cells.

In our experimental model of human zinc deficiency, we observed that NPase activity in lymphocytes was decreased in zinc-deficient subjects and that decreased NPase may partly account for abnormal T cell functions in zinc deficiency [31, 32].

## 5. Zinc and Neutrophils

Briggs et al. in 1982 reported that granulocytes in zinc-deficient uremic subjects showed impaired mobility and decreased both chemotactic and chemokinetic activities, in comparison with subjects who were supplemented with zinc. Others also observed abnormal granulocyte chemotaxis, corrected by zinc supplementation in patients with acrodermatitis enteropathica and in chronic renal disease patients without uremia [33]. Thus, it appears that zinc is essential for chemotaxis.

## 6. Zinc Modulates Cell-Mediated Immune Functions and Participates in T Helper Cell Differentiation

Zinc is essential for cell-mediated innate immunity and activities of natural killer cells. Macrophages are also affected by zinc deficiency. Phagocytosis, intracellular killing, and cytokine production by these cells are affected by zinc deficiency. The growth and function of T and B cells are affected adversely due to zinc deficiency. Zinc is needed for DNA synthesis, RNA transcription, and cell division [25]. Zinc deficiency adversely affects the generation and functions of cytokines, the basic messengers of the immune system [18, 19].

**Figure 1:**
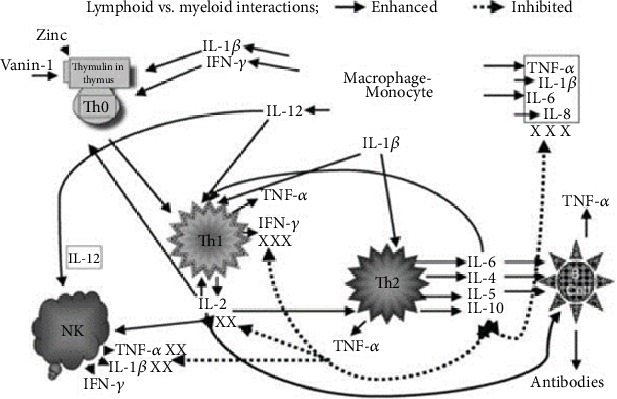
The landscape of zinc action on immune cells. Zinc is an essential component of thymulin, a thymic hormone involved in maturation and differentiation of T cells. The gene expression of IL-2 and IFN-*γ* (Th1 cytokines) is zinc dependent. IL-2 is involved in the activation of NK and T cytolytic cells. IL-12 is generated by stimulated macrophages-monocytes and is zinc dependent. IFN-*γ* and IL-12 together play a major role in the killing of parasites, viruses, and bacteria by macrophages-monocytes. Th2 cytokines are not affected by zinc deficiency except for IL-10 production, which is increased in the zinc-deficient elderly subjects. This is corrected by zinc supplementation. Increased IL-10 affects adversely Th1 and macrophage functions.

Figure 1 shows the landscape of action of zinc on immune cells.

Our studies have shown that serum thymulin activity is zinc dependent [18]. Thymulin, a thymopoietic hormone, is required for proliferation and differentiation of T helper cells. We observed that Th1 (T helper 1) functions in human zinc deficiency were affected adversely. Zinc was also required for gene expression of IL-2 and IFN-*γ* from Th1 cells, and the generation of these cytokines was adversely affected due to zinc deficiency. IL-2 deficiency decreased adversely NK cell lytic activity and decreased T cytotoxic cells [22]. Decreased IFN-*γ* affected macrophages and monocyte function affecting killing of viruses, bacteria, and other infective agents. Zinc deficiency in humans resulted in decreased Th1 functions but did not affect Th2 cells; thus, there was an imbalance between Th1 and Th2 functions, resulting in adverse effect on cell-mediated immunity.

Zinc deficiency resulted in activating monocytes-macrophages, which resulted in increased generation of inflammatory cytokines such as TNF-*α*, IL-1*β*, and IL-6 and increased oxidative stress [34].

**Figure 2:**
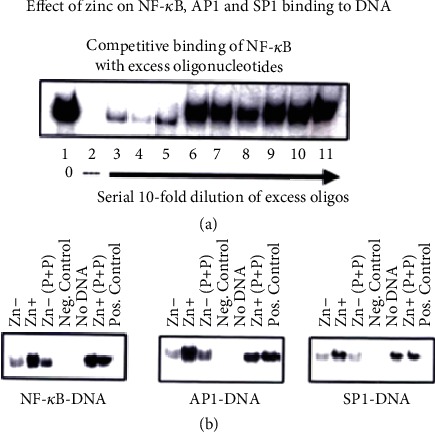
In a cell culture model HUT-78, the binding of NF-*κ*B to DNA, an essential transcription factor for gene expression of Th1 cytokines (IL-2 and IFN-*γ*), is regulated by zinc as shown in this figure. We also show that the other transcription factors essential for gene expression of Th1 cytokines, AP1 and SP1, are also zinc dependent and their binding to DNA is regulated by zinc [35].

Figure 2 shows the effect of zinc on NF-*κ*B, AP1, and SP1 binding to DNA. Zinc deficiency decreases the binding of NF-*κ*B, AP1, and SP-1 to DNA [35].

**Figure 3:**
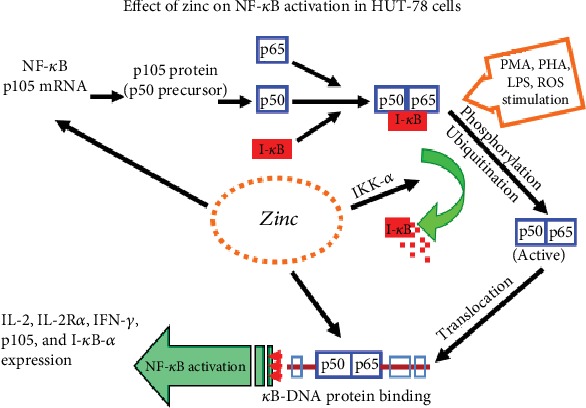
Our results of activation of NF-*κ*B by zinc in HUT-78, a cell culture model. We observed that zinc was required for the expression of p105 mRNA, a precursor of p50 NF-*κ*B protein. Once expressed, p50 NF-*κ*B binds to I*κ*B in the plasma. Following phosphorylation of I*κ*B by zinc, NF-*κ*B 50 is released for binding to DNA and gene expression of various proteins such as IL-2, IL-2R*α* and *β*, IFN-*γ*, and I*κ*B-*α*.

Figure 3 summarizes the effects of zinc on NF-*κ*B activation in HUT-78 cells. Zinc is involved at several steps in the activation of NF-*κ*B in HUT-78 cells. Zinc is required for gene expression of NF-*κ*B and its activation.

**Figure 4:**
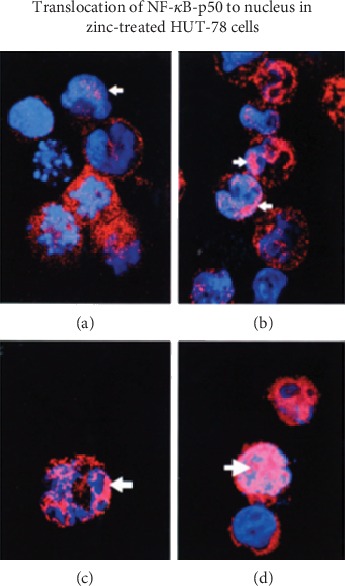
The translocation of NF-*κ*B to DNA for binding and gene expression. Translocation of NF-*κ*B-p50 to the nucleus under zinc-deficient and zinc-sufficient conditions. HUT-78 cells were incubated under Zn- and Zn+ conditions for 4 days and then exposed to PMA/PHA for 3 hours. Confocal images were prepared to show cytosolic NF-*κ*B and nuclear material and the colocalization of NF-*κ*B in the nucleus: (a) nonstimulated Zn- cells; (b) nonstimulated Zn+ cells; (c) PMA/PHA-stimulated Zn- cells; (d) PMA/PHA-stimulated Zn+ cells. Arrows indicate areas of colocalization. PMA/PHA-stimulated Zn+ cells showed the greatest translocation of NF-*κ*B-p50 to the nucleus [36].

Figure 4 shows our experiment in HUT-78 cells. HUT-78 (Th0) cells were incubated under zinc-deficient and zinc-sufficient conditions for 4 days and then exposed to PMA/PHA for 3 hours. This figure shows confocal images of cytosolic NF-*κ*B and nuclear NF-*κ*B. This figure shows that PMA/PHA-stimulated Zn+ cells showed greater localization of NF-*κ*B to nuclear DNA compared to zinc-deficient cells [36].

**Figure 5:**
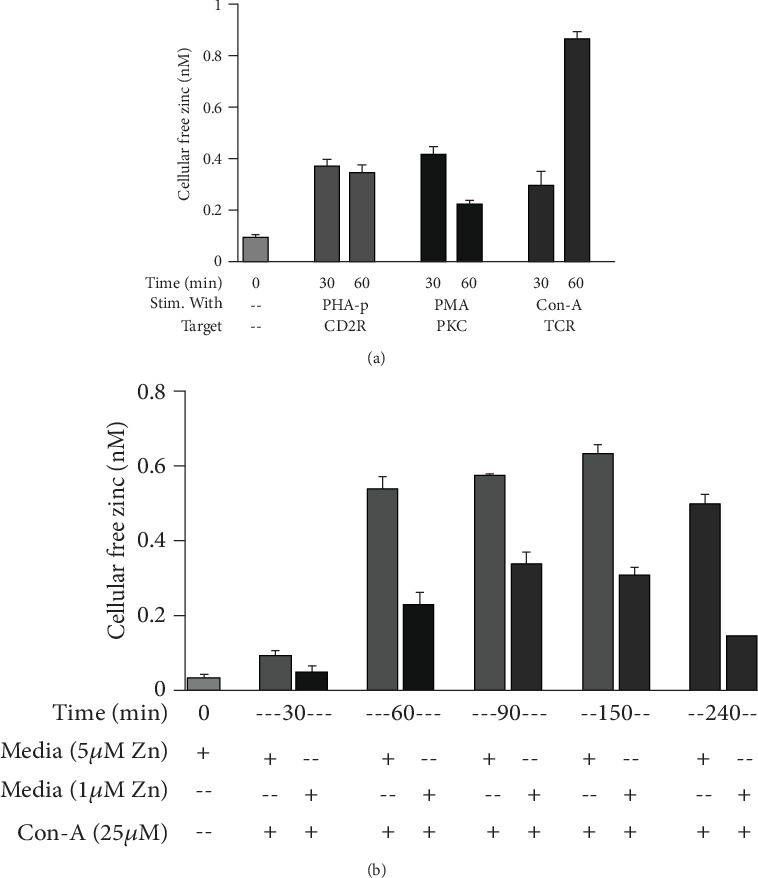
Activation of TCR increases intracellular free zinc. (a) Cells were incubated for 24 h in normal media to which had been added 15 *μ*M zinc (to load cells with zinc) and then stimulated in normal media (5 *μ*M zinc) in the presence of PHA, PMA, or Con-A for 30 and 60 min to determine which pathway was involved in differentiation or activation of Th0 or naïve cells and resulted in an increase in cellular free zinc. (b) To determine the time course for an increase in cellular free zinc, HUT-78 cells were stimulated in normal or in zinc-deficient medium. Stimulation in normal vs. zinc-deficient media allowed us to determine if the increase in cellular free zinc was due to an influx of extracellular free zinc or only a release of free zinc within the cellular compartment under Con-A stimulation. It appears that the increase in cellular free zinc is a result of both extracellular zinc influx and the release of free zinc from intracellular sources following Con-A stimulation (*n* = 3) [37].

Figure 5 shows that intracellular free zinc increased in HUT-78 (Th0) cells following Con-A stimulation of cells via TCR receptor, releasing intracellular free zinc which functions as a signal molecule for generation of IFN-*γ*, T-bet, and IL-12R*β*2, mRNAs required for Th1 cell differentiation [37].

**Figure 6:**
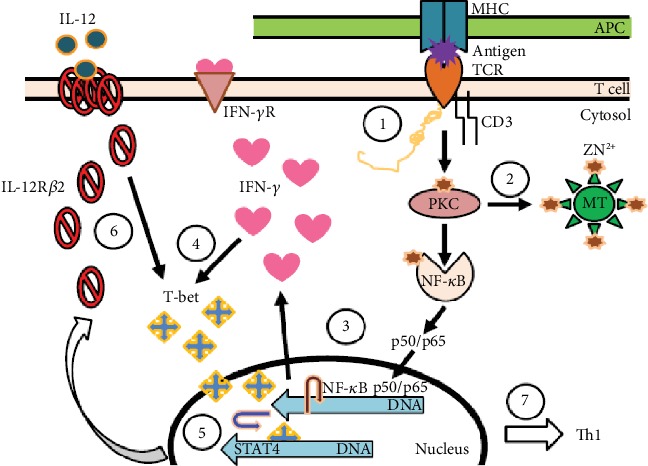
Role of zinc in differentiating Th0 T cells to the Th1 subtype. The differentiation of Th0 or CD4+ naïve T cells to the Th1 subtype is a two-stage process involving many transcription factors and events which are zinc-dependent. (1) Engagement of TCR by CD3 antibodies or Con-A or an antigen by APC is the first step in the differentiation process. Here, zinc acts as a bridge between the CD4 or CD8 receptor and Lck, a tyrosine kinase activated during differentiation. (2) Once the initial process begins, zinc-dependent PKC-h is phosphorylated and able to activate the release of free zinc from metallothionein, the endoplasmic reticulum, or the Golgi. (3) An increase in free zinc is then used for binding of activated NF-*κ*B-p50/p65 to DNA, which initiates the transcription and production of IFN-*γ*. (4) IFN-*γ* enhances the expression of T-bet. IFN-*γ* and T-bet expression then becomes autocrine/paracrine. During the second stage, after TCR is disengaged. (5) T-bet associates with STAT4 for transcription and expression of IL-12R*β*2 which is a zinc-dependent process. (6) Once the expression of IL-12R*β*2 is enhanced, T-bet expression then depends upon IL-12. (7) Once the Th1 cells are differentiated and stabilized, they function in the absence of IFN-*γ*. Full expression of T-bet also inhibits the expression of GATA3 which is responsible for Th2 differentiation, thus, locking in the Th1 phenotype [37].

Figure 6 shows schematically the role of various factors involved in Th1 cell differentiation. The increase in intracellular free zinc follows Th0 stimulation with Con-A, which functions as the upregulator of various transcription factors. It upregulates IFN-*γ*, T-bet, and IL-12R*β*2 receptors and STAT4 for Th1 cell differentiation [36].

## 7. Zinc and Allergy

In one study, the influence of zinc on allergen-induced cell growth, CD4+ regulatory T (Treg) cells, and cytokine expression during allergic immune reaction was investigated [38].

PBMC from nonatopic and atopic subjects were treated with timothy green allergen preincubated with or without (50 *μ*M) zinc.

In CD3+ T cells, combination of zinc 50 and allergen significantly reduced PBMC proliferation of atopic subjects. Zinc 50 plus allergen enhanced Th1 cytokines as shown by increased IFN-*γ*/IL-10 ratios, and enhanced generation of Treg cells was observed. Zinc appeared to downregulate unwanted hyperresponsive cells. There was a significant shift from IL-10 to Th1 cytokine IFN-*γ* and enhanced generation of Treg cells.

This study suggests that zinc supplementation may be useful as a therapy for allergies without negatively affecting the immune system.

T lymphocytes regulate and coordinate immune responses in allergic diseases. Treg cells suppress CD4+ effector T cells, CD8+ T cells, antigen-presenting cells (APCs), NK cells, and also B cells. A decreased expression of Tregs was observed in patients with asthma and allergic rhinitis, and this results in expansion of Th2 cells [39].

Allergies belong to the Th2-driven diseases and are often accompanied by zinc deficiency and are therefore targets for zinc-induced modulation of the allergic immune reactions [40, 41].

Th1 and Th17 cells also contribute to allergic inflammation and hyperresponsiveness [42–44].

Treg inhabits T cell activation following allergen exposure, but this process is not optimal in atopic individuals.

Allergic asthmatic patients have impaired Treg-mediated suppression, and this correlates well with decreased serum zinc levels as reported in many studies.

Zinc supplementation is able to restore Treg function. Zinc deficiency in pregnancy is known to increase the incidence of allergies in children [45].

## 8. Zinc Decreases Oxidative Stress and Decreases the Generation of Chronic Inflammatory Cytokines in Humans

**Figure 7:**
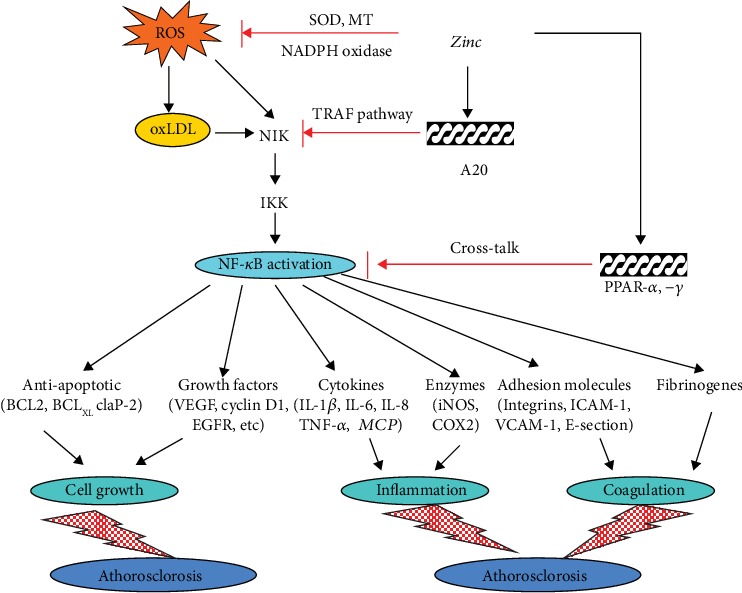
Our concept regarding the role of zinc as an antioxidant and anti-inflammatory agent. Reactive oxygen species (ROS) is known to activate NF-*κ*B. Zinc decreases ROS generation. NADPH oxidase is inhibited by zinc and SOD, which is both a zinc and copper-containing enzyme that is upregulated. SOD is known to decrease oxidative stress. Metallothionein (MT) is induced by zinc and MT, which contains 26 moles of cysteine per mole of protein, decreasing OH burden. Zinc via A20 inhibits NF-*κ*B activation, and this results in a decrease in generation of inflammatory cytokines and adhesion molecules. This figure also shows that zinc may have a preventive role in some cancers such as colon and prostate and in atherosclerosis inasmuch as chronic inflammation has been implicated in the development of these disorders [46, 47].

Figure 7 summarizes our concept regarding the role of zinc as a proantioxidant and anti-inflammatory agent.

ROS is known to activate NF-*κ*B. Zinc decreases NADPH oxidase activity, and SOD is a zinc-copper-containing enzyme which is upregulated by zinc. These effects of zinc decrease oxidative stress. Zinc also upregulates MT synthesis, and MT decreases ^·^OH burden.

Zinc upregulates A20, a transcription factor which inhibits NF-*κ*B activation [34, 46].

Downregulation of NF-*κ*B decreases generation of inflammatory cytokines which decreases ROS.

The anti-inflammatory role of zinc may have protective effect on atherosclerosis, prostate cancer, and colon cancer.

## 9. Effect of Marginal Zinc Deficiency in Human on Sensitive Immunological Parameters

Zinc deficiency increases oxidative stress and upregulates generation of inflammatory cytokines in humans [46].

Zinc supplementation decreases NADPH oxidase in monocytes-macrophages, which decreases generation of free radicals. Superoxide dismutase is both a zinc- and copper-dependent enzyme, which is essential for downregulating free radicals. Metallothionein is a zinc protein very effective in decreasing ^·^OH; thus, zinc is an effective agent to decrease oxidative stress. Zinc supplementation increases A20 (a zinc-dependent transcription factor) activity in monocytes-macrophages, and A20 downregulates generation of TNF-*α*. Thus, zinc is an effective agent in decreasing oxidative stress and decreasing generation of inflammatory cytokines [46, 47].

**Figure 8:**
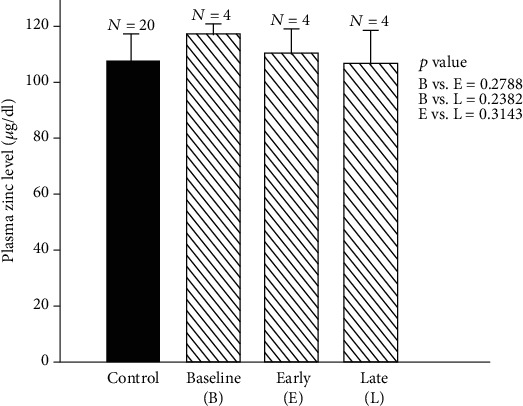
Changes in plasma zinc during early and late zinc deficiency periods in marginal deficiency of zinc in humans. Plasma zinc levels (mean ± SD) during baseline (B) vs. early zinc deficiency period (E) and late zinc deficiency period (L) were as follows: B vs. E, 116.20 ± 3.51 *μ*g/dl vs. 109.10 ± 8.30 *μ*g/dl, *p* = 0.27; B vs. L, 116.20 ± 3.51 *μ*g/dl vs. 105 ± 11.38 *μ*g/dl, *p* = 0.23; and E vs. L, 109.10 ± 8.30 *μ*g/dl vs. 105.53 ± 11.38 *μ*g/dl, *p* = 0.31. The values for plasma zinc in normal control subjects (mean ± SD) are also shown (107.26 ± 8.92 *μ*g/dl) [48].

Figure 8 shows the effect of marginal zinc deficiency in humans on plasma zinc [48].

**Figure 9:**
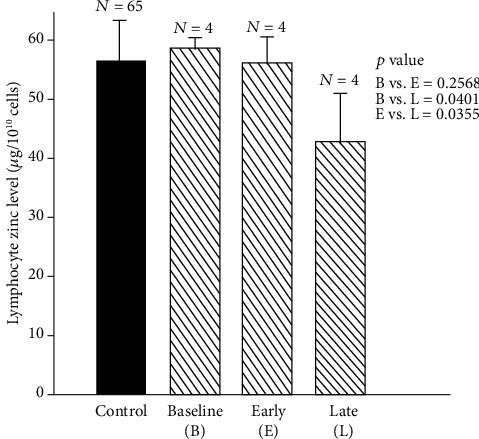
Changes in lymphocyte zinc level during early and late zinc deficiency periods in the experimental model of human zinc deficiency. Lymphocyte zinc levels ((mean ± SD) *μ*g/10^10^ cells) during baseline (B) vs. early zinc deficiency period (E) and late zinc deficiency period (L) were as follows: B vs. E, 58.36 ± 1.64 *μ*g/10^10^ cells vs. 55.29 ± 4.20 *μ*g/10^10^ cells, *p* = 0.25; B vs. L, 58.36 ± 1.64 *μ*g/10^10^ cells vs. 41.67 ± 8.26 *μ*g/10^10^ cells, *p* = 0.04; E vs. L, 55.29 ± 4.20 *μ*g/10^10^ cells vs. 41.67 ± 8.26 *μ*g/10^10^ cells, *p* = 0.03. The lymphocyte zinc level (mean ± SD) for control subjects is also shown (56.56 ± 6.42 *μ*g/10^10^ cells [48].

Figure 9 shows the effect of marginal zinc deficiency in the experimental model [48].

**Figure 10:**
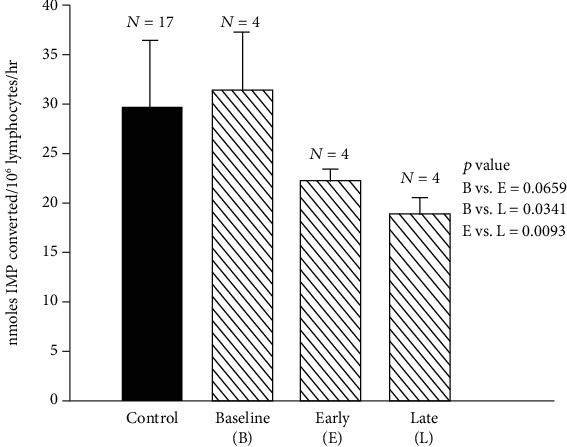
Changes in lymphocyte 5′NT activity during baseline, early zinc deficiency, and late zinc deficiency periods in the experimental model of human zinc deficiency IL 5′NT activity (mean ± SD) nmoles IMP converted/10^6^ lymphocytes/hour) during baseline (B) vs. early deficiency period (E) and late deficiency period (L) were as follows: B vs. E, 31.13 ± 5.56 nmol IMP converted per 10^6^ lymphocytes per hour vs. 21.95 ± 0.92 nmol IMP converted per 10^6^ lymphocytes per hour, *p* = 0.06; B vs. L, 31.13 ± 5.56 nmol IMP converted per 10^6^ lymphocytes per hour vs. 18.50 ± 1.58 nmol IMP converted per 10^6^ lymphocytes per hour, *p* = 0.03; E vs. L, 21.95 ± 0.92 nmol IMP converted per 10^6^ lymphocytes per hour vs. 18.50 ± 1.58 nmol IMP converted per 10^6^ lymphocytes per hour, *p* = 0.009. The values for 5′NT in normal control subjects are also shown (29.5 ± 6.53 nmol IMP converted per 10^6^ lymphocytes per hour) [48].

Figure 10 shows the assay of ecto-5′-nucleotidase, an enzyme that is a marker of lymphocyte maturity. Ecto-5′NT is a very sensitive biomarker of human marginal zinc deficiency [48].

**Figure 11:**
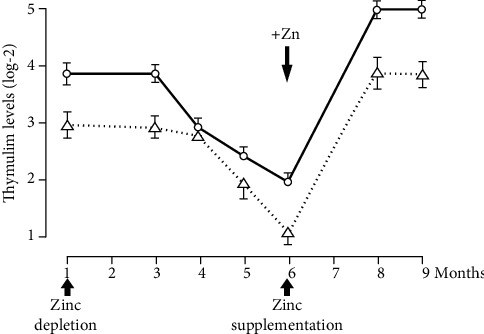
Thymulin activity—levels of thymulin activity in a sequential study of young human volunteers submitted to a zinc-restricted diet for 6 mo followed by zinc supplementation are shown here. Results are expressed as log-2 reciprocal titers (mean ± SEM). Each determination was performed in triplicate [18].

Figure 11 shows the serum active thymulin (thymic hormone) in marginal human zinc deficiency. This also illustrates that the assay of active serum thymulin may be a very good biomarker of marginal human zinc deficiency [18].

Our studies have documented that the assay of immunological parameters may be more sensitive biomarkers of marginal zinc deficiency in humans than the assay of zinc in plasma or blood cells. Serum thymulin activity, IL-2 mRNA generation after PHA-PMA activation of peripheral blood mononuclear cells, and assay of activity of ecto-5′NT, a marker for lymphocyte maturity, are more sensitive biomarkers of zinc deficiency in comparison to the assay of zinc in plasma or peripheral blood cells.

## 10. Conclusion

Zinc deficiency is very prevalent worldwide. Cell-mediated immune dysfunction leading to increased infection is common in zinc-deficient subjects. Zinc is essential for the serum thymulin activity. Thymulin, a thymopoietic hormone, is essential for proliferation of Th0 cells. T-bet (a zinc-dependent transcription factor), INF-*γ*, and STAT4 are required for differentiation of Th1 cells from Th0 cells. Zinc-dependent transcription factors, NF-*κ*B, AP1, and SP-1 are required for generation for IL-2 mRNA from Th1 cells. Zinc deficiency in humans results in downregulation of IL-2 cytokine from Th1 cells. IL-2 is essential for NK cell lytic activity and activation of T cytolytic cells, which participate in killing of viruses, bacteria, and cancer cells.

In zinc deficiency, although Th1 cells are affected adversely, Th2 cells remain unaffected. This results in a shift from Th1 to Th2 functions and results in cell-mediated immune dysfunction in zinc deficiency.

Zinc deficiency in humans activates monocytes-macrophages, which generate free radicals leading to oxidative stress and upregulate generation of inflammatory cytokines such as TNF-*α*, IL-1*β*, and IL-6. Inasmuch as many chronic diseases in humans including cancer of the prostate and colon and atherosclerosis have been related to increased oxidative stress and chronic inflammation, zinc supplementation to decrease oxidative stress and inflammatory cytokines may prove to be very useful in management of a few chronic diseases.

In our studies in the experimental model of mild human zinc deficiency, we observed that serum thymulin activity declined within eight to twelve weeks of institution of zinc-deficient diet in human volunteers [18]. At the same time, we observed that IL-2 mRNA and IL-2 generation from lymphocytes decreased the lymphocyte zinc decreased at the end of twenty weeks, and the decrease in plasma zinc was noted at the end of 24 weeks [48]. These observations suggested to us that the assay of immunological parameters was more sensitive than the assay of zinc in plasma and peripheral blood cells.

We published later that the assay of IL-2 mRNA may be a specific and sensitive biomarker of human zinc deficiency [49].

Immunological parameters such as assays of thymulin activity and lymphocyte ecto-5′-nucleotidase, a marker of lymphocyte maturity, and determination of mRNAs of IL-2 and IFN-*γ* after PHA and PMA stimulation of mononuclear cells in peripheral blood may be very sensitive and useful biomarkers of human zinc deficiency. These assays are more sensitive than determination of zinc in plasma, lymphocytes, neutrophils, or platelets.

## Abbreviations

A20: Zinc-containing transcription factor
DNA: Deoxyribonucleic acid
HUT-78: Human malignant lymphoblastoid cell line of Th0 phenotype
ICAM-1: Soluble intercellular adhesion molecule-1
IFN-*γ*: Interferon-*γ*
I*κ*B: Inhibitory protein of NF-*κ*B
IL-1*β*: Interleukin-1*β*
IL-2: Interleukin 2
IL-6: Interleukin 6
IL-10: Interleukin 10
IL-12R*β*2: Interleukin 12 receptor, beta 2
mRNA: Messenger RNA
MT: Metallothionein
MyD88: Myeloid differentiation primary response 88
NADPH: Nicotinamide adenine dinucleotide phosphate
NF-*κ*B: Nuclear factor kappa B
NK cell: Natural killer cells
PHA: Phytohemagglutinin-p
PMA: Phorbol myristate acetate
RNA: Ribonucleic acid
ROS: Reactive oxygen species
SOD: Superoxide dismutase
STAT4: Transcription factor STAT4
T-bet: Transcription factor involved in T cell differentiation
T cell: Thymic-dependent cell
TCR: T cell receptor
Th: T helper cell
Th1: T helper one
TNF-*α*: Tumor necrosis factor-*α*
TRIF: Domain containing adapter-inducing interferon-*β*
TTP: Thymidine triphosphate
VCAM-1: Soluble vascular cell adhesion molecule
WHO: World Health Organization
ZIP-4: SLC 39 a solute carrier.
